# Supplementary material for: Are carbon emissions trading and green financial instruments synergistic? -Comprehensive quantitative research based on content analysis
Source: PLoS One. 2024 Mar 7;19(3):e0298601. doi: 10.1371/journal.pone.0298601 (PMC10919601; doi:10.1371/journal.pone.0298601)
Supplement: S1 Data — (ZIP) [file pone.0298601.s001.zip › Data/地级市Z_F工作报告.zip!/╝¬┴╓/░╫╔╜╩╨/└┤╫╘╣1⁄2╓┌║┼í╛┬φ┐╦╩2╛▌═°í┐_2019.docx]

各位代表：

　　现在，我代表市政府，向大会作政府工作报告，请予审议，并请市政协各位委员提出意见。

　　一、2018年工作回顾

　　过去的一年，是白山改革发展进程中具有十分重要意义的一年。全市上下深入学习贯彻习近平新时代中国特色社会主义思想、党的十九大以及习近平总书记视察东北期间重要讲话和重要指示精神，在省委、省政府和市委坚强领导下，接受了中央脱贫攻坚专项巡视、中央环保督察“回头看”、国务院大督查、省扫黑除恶专项斗争调研指导等政治检验，坚定信心、保持定力、克难奋进、扎实工作，绿色转型全面振兴高质量发展迈出坚实步伐。

　　一是坚定不移落实新发展理念，经济发展稳中向好。积极应对经济下行压力，经济增长从主要依靠工业带动转为一二三产共同带动。预计全年GDP增长3%，规上工业增加值增长2.3%，服务业增加值增长3.9%，社会消费品零售总额增长5.2%，实现一般公共预算全口径财政收入50.1亿元，地方级财政收入22.7亿元，一般公共预算支出191.6亿元。工业筑底回升。实施工业经济提振行动计划，对规上企业一企一策、精准包保，对特困企业实施“四个一批”，28户“双停”企业复产或得到有效处置，板石矿业、琦祥纸业等50户重点企业总产值增长6.7%。逐个破解工业项目建设滞缓、半截子工程问题，鼎运新能源120万吨焦化产能等量替代被列入国家工业节能与绿色发展重点项目，开复工建设千万元以上工业项目142个，73个项目列入省转型升级重点项目库，完成工业技改投资56.4亿元，增长8.1%。农业结构优化。大力推进农业种植结构调整，调减籽粒玉米14.6万亩，新增特色种植9.3万亩，高效作物占比达60%。扎实推进农村一二三产融合发展，加快建设靖宇、江源国家农村产业融合发展示范园。新培育长白山寒地蓝莓等3个国家农产品区域公共品牌。与京东集团签订战略合作协议，开创地级市与京东合作先河。实现特产业产值155亿元，增长9%，粮经菜比例调整为5:4:1。服务业提档升级。衡睿车城等40个重点服务业项目开工，怡康医养结合综合体主体完工。深入开展“促消费、稳增长”攻坚行动，新增限上批零住餐企业25户，省级电商示范村镇12个。全市电商零售额实现21亿元，增长40%，新业态成为拉动服务业增长新引擎。

　　二是坚定不移推动新旧动能转换，发展质量和效益不断提升。落实高质量发展要求，推动经济转型升级、提质增效。产业升级步伐加快。我市被授予“中国硅藻土之都”称号，成功举办全国矿物复合材料学术会议。施慧达药业完成新厂主体建设，再次登上“健康中国·品牌榜”，入选中国医药工业最具成长力企业十强。万良国家级人参市场正式开市。泉阳泉成为南航航班饮用水中标产品及2018年中非合作论坛北京峰会用水。成功举办“长白山之夏”“长白山之冬”旅游节，白山入围“国内10大长线游”热门目的地城市，全市接待国内外游客1200万人次，实现旅游社会总收入190亿元，分别增长13%和20%。“新五样”占GDP和财政收入比重上升到65.2%和55.2%。项目建设扎实推进。开展“冬季三项促投资”和项目大巡检，开复工5000万元以上项目161个，农夫山泉200万吨饮用水、卫健降脂饼干等31个项目竣工投产。大力实施招商引资“冬春会战”，市级领导带队赴京沪苏粤等地开展精准招商，中节能、东北亚创融中心、中展创投等一批大项目落户白山。民营经济发展壮大。加快推进东北地区首批民营经济改革发展示范市建设，启动“双百”“小升规”等专项行动，市双创孵化基地投入运营，31户民营企业进入新三板、主板上市培育期。全市民营企业和个体工商户分别达到1.4万户和8.2万户，增长15%和9.8%。民营经济对GDP、财政收入和就业贡献率分别达到54.5%、73.6%和70%。

　　三是坚定不移推进改革开放创新，发展活力和内生动力不断增强。坚持用改革的办法、开放的视野、创新的举措，为发展添活力、增动力。改革全面深化。“三去一降一补”扎实推进，煤炭去产能215万吨。“放管服”和“只跑一次”改革成效显著，在全省率先推行“一承诺、双体验、再评价”模式，全省最大政务大厅投入使用，首家行政审批局在临江挂牌，我市不动产登记窗口入选“全国百佳不动产登记便民利民示范窗口”，实现行政村和社区便民服务中心全覆盖。金融、农村等领域改革稳步推进。开放不断扩大。编制完成鸭绿江500公里开发开放经济带总体规划，完成白山经开区新区双创孵化基地及一期道路征地和可研编审。临江边境经济合作区和白山海关获批，长白口岸通过国家一类口岸验收。与湖州对口合作不断深化，签订25个战略合作协议。创新能力增强。大力开展“百所高校进白山”“院士专家白山行”等活动，出台人才工作“一个意见、九个方案”，引进高层次人才104名。新增和一硅藻科技等9户国家高新技术企业、长白山药业等2户国家知识产权优势企业、12户省科技小巨人企业。实施35个科技成果转化项目，31个省级创新项目获批。

　　四是坚定不移推进区域协调发展，城乡发展新格局逐步形成。坚持以城市建设为引领、美丽乡村为基础、交通路网为支撑，推动城乡一体化发展。城市建设“提质”。成功举办中国·白山城市规划研讨会，进一步明确城市发展方向。大力推进市区带状公园、北山公园改造等39个城建项目，滨江北街等5条断头路全部打通。江源老旧城区改造、长白街路广场、靖宇刘海山休闲广场、抚松西江公园等项目顺利实施，临江入选中国城市品牌（县级市）百强榜。美丽乡村“提标”。全面启动农村人居环境整治三年行动，创建省级美丽乡村示范村23个，美丽庭院和干净人家星级文明户各1000户，实施农村安全饮水工程129处，68%行政村实现垃圾“村收集、镇转运、县处理”。临江火绒沟村和转头山屯入选第五批中国传统村落名录。大交通体系“提能”。辉白高速竣工通车，结束市区不通高速历史。白山机场完成选址，沈白高铁建设正式启动。积极推进白临、松长高速建设。白山至三道沟公路竣工通车，三道沟至错草、靖宇至江源、松江河至泉阳公路启动建设。

　　五是坚定不移践行绿色发展理念，生态文明建设和环境保护成效明显。以最坚决的态度、最迅速的行动、最有力的举措，全力配合中央环保督察“回头看”和省级环保督察，高效办结749件交办案件，超常举措完成长白山国际度假区高尔夫球场和别墅问题整改。开展市级常态化生态环保督察。加大环境违法犯罪打击力度，成立全省首个公安生态环境犯罪侦查支队。深入开展大气、水、土壤污染防治攻坚战。全部完成20蒸吨及以上燃煤锅炉改造，淘汰黄标车和老旧车辆2986台，市区空气优良天数突破350天，环境空气质量达到国家二级标准。全面落实河长制，水环境质量全省第一，9处县级以上饮用水源水质达标率100%。江源餐厨垃圾处理项目主体基本封闭，市生活垃圾处理场整改修复工程加快推进，南山垃圾处理场完成雨污分流工程。禁养区内72家养殖场全部关闭搬迁。实施山水林田湖草生态修复治理一期工程，开展自然保护区、矿区、水源保护区生态环境综合整治“回头看”，完成森林抚育79.6万亩。长白、临江、抚松入选2018全国百佳深呼吸小城，长白连续两年荣登榜首，吸引众多媒体关注聚焦，被誉为“中国最美小城”。

　　六是坚定不移贯彻以人民为中心的发展思想，民生福祉进一步改善。始终坚持新增财力80%以上用于民生，预计城乡居民人均可支配收入分别增长3%、5.5%。脱贫攻坚取得实效。制定《打赢脱贫攻坚战三年行动实施意见》，实施111个产业扶贫项目和170个基础设施扶贫项目。狠抓脱贫攻坚历次巡视、暗访、督查考核反馈问题整改工作。推进扶贫审计全覆盖，审计问题整改率达100%。全市减贫5304人，退出贫困村56个，抚松荣获“首批全国脱贫攻坚优秀城市”称号。保障体系不断完善。实施全民参保计划，实现社保“一卡通”全覆盖。累计救助城乡困难群体12.6万人次，发放救助金和低保金5亿元，城乡低保标准分别提高到月人均540元和年人均3600元。开展“慈善光明行”活动，治愈贫困白内障患者300名。实施“创业引领”“就业扶持”工程，城镇新增就业3.8万人，城镇登记失业率控制在3.7%，农村劳动力转移就业10.6万人。社会事业全面发展。新改扩建14所幼儿园，启动外国语学校扩建工程，长白山职业技术学院新校区投入使用。合理配置医疗资源，深化城市公立医院改革，建成全科医生培训基地和康健医院，县域内就诊率达90%以上，我市被评为全国“平安医院”创建先进地区。长白山文创园成功申报省级文化产业园，纪念改革开放40周年献礼电影《中国年》在临江完成拍摄，江源广电台入选改革开放40年全国百佳县级广电台。全民健身、竞技体育和体育产业协调发展，取得省级以上赛事奖牌179块。社会保持和谐稳定。全面开展扫黑除恶专项斗争，实施“天网”“地网”安全防范工程，扎实推进基层平安建设，刑事案件发案率下降21.7%，连续7年实现现行命案100%侦破。开展“安全生产治理年”活动和十大专项治理工程，连续33个月无重大安全事故。深刻汲取长生生物问题疫苗案件教训，不断完善食品药品监管体系，未发生系统性、区域性重大食品药品安全突发事件。有效抵御“温比亚”“苏力”等台风灾害。连续39年无重大森林火灾。推进信访维稳百日攻坚行动和重点信访案件攻坚年活动，着力解决9.5万户661.8万平方米“无籍房”等突出问题，信访案件总量下降22.6%。持续深化党政军警民合力治边，边境形势总体平稳。加强民族工作，依法管理宗教事务，举办长白60周年县庆系列活动，长白县和白山方大集团入选第六批全国民族团结进步创建示范区（单位）。

　　七是坚定不移坚持党的领导，政府治理能力和水平进一步提升。认真落实全面从严治党要求，扎实推进“两学一做”学习教育常态化制度化。全面开展政府系统干部作风大整顿，驰而不息查“八不”、破“五弊”、纠“四风”。开展解放思想推动高质量发展大讨论活动，营造勇于革故鼎新、锐意攻坚克难、奋力开拓进取的浓厚氛围。不断加强政府系统廉政建设和反腐败斗争，行政监察、审计监督力度进一步加大，全年行政问责206人，查处发生在群众身边的不正之风和腐败问题263起，处理268人。主动接受各方监督，提请市人大常委会审议地方性法规草案1部，办理人大代表建议和政协委员提案117件，办结率100%。强化依法行政，修订完善《政府工作规则》，建立健全科学民主决策、政务公开等14项制度。

　　各位代表，过去一年的成绩来之不易。这是以习近平同志为核心的党中央统揽全局、科学决策的结果，是市委团结带领全市人民万众一心、奋力拼搏的结果，是市人大、市政协有效监督、大力支持的结果。在此，我代表市政府，向全市各族人民，向各民主党派、工商联、人民团体和社会各界人士，向驻白人民解放军、武警部队官兵和公安干警，向所有关心、支持、参与白山振兴发展的海内外朋友，表示崇高敬意和衷心感谢！

　　在肯定成绩的同时，我们也清醒看到面临的困难和问题。经济企稳回升基础仍不牢固，结构不优、质量效益不高的状况没有得到根本改变，传统产业动能减弱、新兴产业成长乏力、新旧动能青黄不接的问题没有得到根本解决；各类风险矛盾交织，防范化解压力大；城乡基础设施和人居环境依然薄弱，民生领域存在不少短板，脱贫攻坚任务艰巨；政府治理体系和治理能力与新时代新要求还不相适应，少数政府工作人员不想为、不会为、不敢为以及工作失职失责失察、不严不实不深不细等作风问题仍然存在。我们将始终保持清醒头脑，坚定发展信心，增强忧患意识，坚持底线思维，加强宏观思考，保持发展定力，推动白山发展走得更稳、走得更远。

　　二、2019年工作安排

　　今年是建国70周年，是全面建成小康社会、加快推动白山绿色转型全面振兴高质量发展的关键之年。放眼国际，尽管单边主义、贸易保护主义抬头，但世界经济总体呈现逐步复苏态势；纵观国内，尽管经济发展面临一些新问题新挑战，下行压力加大，但总体平稳、稳中有进，经济结构持续优化，主要宏观调控指标处于合理区间；环视周边，尽管东北亚地区局势还存在变数，但半岛形势总体趋稳，白山区位优势得以显现，知名度和关注度进一步增强；审视自身，随着“大交通”体系日益完善，特别是未来高铁建成通车，既为我们提供了抢占先机、加速崛起的战略机遇，也带来了潜在的虹吸效应、遮阴效应和屏障效应，使我市面临被进一步边缘化的可能。我们必须未雨绸缪、抢占先机，下苦功夫、下实功夫、下硬功夫，强弱项、补短板、扬优势，以“宝剑锋从磨砺出，梅花香自苦寒来”的精神风貌，迎接高铁时代到来。

　　今年政府工作总体要求是：高举习近平新时代中国特色社会主义思想伟大旗帜，认真贯彻党的十九大和十九届二中、三中全会以及中央、全省经济工作会议精神，全面落实习近平总书记对东北振兴工作重要指示精神，按照市委七届五次全会暨全市经济工作会议部署，统筹推进“五位一体”总体布局，协调推进“四个全

　　面”战略布局，坚持稳中求进工作总基调，坚持新发展理念，坚持推动高质量发展要求，坚持以供给侧结构性改革为主线，坚持生态立市、产业强市、特色兴市，深入实施“3331”发展战略，继续打好“三大攻坚战”，统筹推进稳增长、促改革、调结构、惠民生、防风险工作，保持经济持续健康发展和社会大局稳定，提高人民群众获得感、幸福感、安全感，奋力开创新时代白山绿色转型全面振兴高质量发展新局面，以优异成绩迎接新中国成立70周年。

　　全市经济社会发展主要预期目标是：GDP增长6%，地方级财政收入与上年持平，规上工业增加值增长5%，服务业增加值增长8%，社会消费品零售总额增长6%，城乡居民人均可支配收入与经济增长同步，城镇登记失业率控制在4.4%以内，单位GDP能耗下降3%以上。

　　上述目标的确定，充分体现了高质量发展与稳中求进的要求，既与上级安排部署相衔接，又有利于确保经济运行在合理区间，实现更高质量、更有效率、更加公平、更可持续的发展；提出财政收入与上年持平，充分兼顾了宏观经济形势与我市发展实际，特别是针对中央将实施更大规模的减税降费，更加切实可行，具有可操作性；提出经济增速略高于全省平均水平，充分考虑了强化社会预期引导，通过释放积极信号，稳定预期，增强市内外各界支持、参与、投身白山绿色转型全面振兴高质量发展的信心和决心。

　　围绕上述目标，重点做好以下七方面工作：

　　（一）聚焦构建绿色产业体系，推动经济发展高质量。有效打通“绿水青山”“冰天雪地”和“金山银山”之间的通道，加快推动质量变革、效率变革、动力变革。

　　强力推进产业转型升级。坚持一二三产融合发展，着力构建多点支撑、多业并举、多元发展的产业格局。以绿色有机激发新动能。围绕参、蛙、菌、药、菜、牧、果、蜂、渔九大特色板块，坚持质量兴农、绿色兴农、品牌强农，建设中国绿色有机谷·长白山森林食药城。持续推进“退粮进特”和“一增一减”，新增特色种植20万亩。实施有机类肥料替代行动，推行测土配方精准施肥和休耕轮作，推广绿色防控技术，加强农产品质量安全追溯体系和品牌建设，在全省率先实现农药化肥施用量“双减半”，新增“三品一标”认证产品5个，创建国家农产品安全示范区。实施新型经营主体培育工程，新增省市级龙头企业8户。推进“互联网+现代农业”，开展电商“双示范”创建。实现特产业产值164亿元，增长5.8%。以资源加工厚植新优势。深入挖掘硅藻土、矿泉水、医药等优势资源潜力，推进精细生产、精深加工、精品营销，加大技术引进和新产品开发力度，延伸产业链条，提升产品附加值，增强核心竞争力。深化与中建材等战略合作，大力推进硅藻土产业整合，创建省级硅藻土产业开发区。鼓励矿泉水企业开发气泡水、母婴水等功能性产品，抢占中高端市场。加快推进施慧达药业新厂、万良人参小镇等项目，支持左氨氯地平比索洛尔片等新药研发，打造中国北药基地和世界人参之都。以全域旅游培育新增长点。编制全域旅游发展规划，设立旅游产业发展基金，加快推进鲁能漫江、泰安湖满族风情园、啤酒小镇等项目，谋划市区至三道沟“百里画廊”等精品线路，串珠成链、串线成面，构建全域旅游新格局。积极发展“旅游+康养”新业态，筹办中国·白山绿色生态康养旅游论坛，打响“山水画卷·生态白山”品牌。做好寒地冰雪文章，加快推进松岭雪村、锦江木屋村等冰雪项目，筹办大型冰雪赛事，打造最美关东雪村。持续放大“长白山之夏”“长白山之冬”节庆活动品牌效应。深入挖掘历史、红色、民俗、生态、边境等文化资源，推进全国青少年长白山革命传统教育基地建设，积极培育文化创意、影视创作等新型文化业态，推动文化和旅游深度融合发展。旅游社会总收入、接待游客人数分别增长15%和12%。以数字信息打造新引擎。深入推进数字白山建设，实施工业互联网建设、新型智慧城市、数字政务增效等十大工程，加快建设长白山云数据基地，推动互联网、大数据、人工智能与实体经济深度融合，运用数字化、网络化、智能化手段改造传统产业、培育新兴产业，促进产业数字化、数字产业化，为经济社会发展插上腾飞翅膀。

　　强力推进项目建设和招商引资。发挥投资关键作用，以优化投资结构助推产业转型升级。着力抓谋划。密切关注国家重点扶持领域和产业发展方向，滚动谋划包装干饭盆生态健康产业园等140个亿元以上项目，争取更多项目、资金和政策落户白山。着力抓引进。持续实施“冬春会战”，完善招商引资大数据库，转变理念、创新方式，既主攻有实力的大企业集团，引进一批引领性、支撑性强的大项目，也突出扩大就业、增加税收，引进一批见效快、成长性好的中小项目，力争签约项目75个。深入实施“白商回归”工程，研究出台重磅优惠政策吸引白山籍企业家返乡创业。着力抓推进。实施“项目建设年”活动，实行重大项目全程秘书式、保姆式服务，突出解决土地、资金等瓶颈，坚决打通影响和制约项目投产达效“最后一公里”。开工建设5000万元以上项目137个，20个项目竣工投产。

　　（二）聚焦打造最优营商环境，推动体制机制高质量。以更大决心和力度深化改革、扩大开放，坚决打好重塑环境攻坚战。

　　大力发展民营经济。深入贯彻习近平总书记在民营企业座谈会上的重要讲话精神，综合施策融化市场“冰山”、削低融资“高山”、跨越转型“火山”。全面放宽市场准入，坚决打破各种“卷帘门”“玻璃门”“旋转门”，拓宽民营企业发展空间。组织开展各类产销对接活动，帮助企业开拓市场。坚持“规定动作”和“自选动作”结合，出台企业降本减负实施意见，能减则减、能免则免，让企业轻装上阵。实施“上市驱动”“引金入白”工程，强化银企对接，组建纾困基金，建设金融集聚区，力争企业直接融资比增长3%。建立健全企业家参与政策制定、联系民营企业等机制，依法保护企业家合法权益，构建“亲”“清”政商关系，当好“店小二”。民营经济主营业务收入增长6%，民营企业和个体工商户数量分别增长7%和5%。

　　持续深化重点领域改革。加快推进“放管服”和“只跑一次”改革，进一步取消和下放审批事项，深化“证照分离”“照后减证”改革，依托新政务大厅打造市级统筹、整体联动、部门协同、一网通办的“互联网+政务服务”体系，力争服务事项全部进厅，削减办事材料60%以上，实现“只进一扇门，能办所有事”。蹄疾步稳推进政府机构等各项改革，确保按期完成任务。

　　全面扩大对外开放。深度融入全省“一主、六双”产业空间布局，加快建设长白国家重点开发开放试验区，力争白山海关正式开关办公，省级矿泉饮品外贸转型升级示范基地获批，长白互市贸易区开工建设，新增外贸主体15户。推动白山经开区新区建设提速，加快建设吉浙对口合作示范园和双创孵化基地，引进10个项目落户。深化与湖州对口合作，开展“湖商白山行”。鼓励企业走出去，积极参加东博会、全球吉商大会等活动。

　　（三）聚焦以创新培育新动能，推动创新体系高质量。围绕创建国家可持续发展议程创新示范区和全省创新型试点市，加快实施创新驱动发展战略。

　　加强创新平台建设。持续深化与高校院所合作，实施30个创新项目，推动10个产学研合作项目落地。开展企业研发平台提质升级行动。深化与湖州“飞地”合作模式，加快建设科创园，共建双创示范基地。

　　加强创新型企业培育。鼓励施慧达、蓝锐等企业走“专精特新”发展道路，力争新培育5户国家高新技术企业、10户省科技小巨人企业。引导更多企业与高校院所共建研发机构，争取规上企业设立研发机构比例达10%以上。强化知识产权创造、保护、运用，实施10个科技成果转化项目。

　　加强创新人才引进培养。持续开展“选拔博士硕士挂职”“院士专家白山行”“百所高校进白山”等活动，引进硕士以上人才50名。依托院士工作站引进院士、博士团队2个，国内外知名专家10位。推进“技能白山”建设，深化产教融合，推动长白山职业技术学院开门办学，与企业共建实训基地，培育适应产业需求的高技能、创新型“白山工匠”。

　　（四）聚焦城乡统筹协调发展，推动城乡环境高质量。坚持全域统筹，加快形成城乡一体化发展新格局。

　　高品质建设生态宜居城市。强化规划引领，大力推进“多规合一”，完善控详规。加快推进长白山大街、浑江大街东延等街路改造项目，打通平阳路等3条断头路。坚持挖潜增绿，打造红旗街等5条特色街路、6个特色游园和绿地。做足水文章，大力推进水系贯通、水体净化、水景提升，基本消除市区黑臭水体。点亮城市夜景，完成满族博物馆等18座城市地标建筑亮化提升工程。完善城市功能，加快推进垃圾处理、城区供水、文体中心等重点工程，新增供热、供水等管网24公里。提升人居环境，改造180栋“老旧散弃”小区、4166户29万平方米棚户区。持续开展交通秩序专项治理。推动物业管理全覆盖、城市管理精细化。

　　高质量建设美丽乡村。深入实施乡村振兴战略，加快推进农村人居环境整治三年行动，统筹推进村容村貌、垃圾污水治理和“四好农村路”建设。下大力气抓好“厕所革命”，探索高寒山区农村卫生厕所维护管理模式。新增2000个美丽庭院和干净人家星级文明户，建设农村公路130公里，自然村屯道路硬化率达到80%。积极吸引社会资本下乡、农村人才留乡、成功人士归乡，培育一批懂农业、爱农村、爱农民的“三农”队伍，引领农业成为有奔头的产业、农民成为有吸引力的职业、农村成为安居乐业的家园。

　　高标准建设大交通体系。完成白山机场预可研，积极配合推动沈白高铁开工建设，全力推进白临、松长高速前期工作，加快建设三道沟至错草、靖宇至江源等干线公路，启动鹤大公路白山绕越线、石通线七道江至通化界及城市综合交通枢纽前期工作。

　　（五）聚焦环境保护和生态文明建设，推动生态环境高质量。巩固提升生态环境比较优势，积极创建国家生态文明建设示范区。

　　全力以赴解决环境突出问题。加大中央和省级环保督察反馈问题整改力度，坚决打好污染防治攻坚战。实施清洁空气行动，重点整治原煤散烧、尾气、扬尘等问题，基本淘汰全市10蒸吨及以下燃煤锅炉，力争市区空气优良天数达到340天。实施清洁水体行动，抓好河流断面水质改善和饮用水源地环境保护，确保“三江”干流水质优良比例保持70%以上。实施清洁土壤行动，强化危险废物源头管理、生活垃圾治理和农业面源污染防治，基本完成自然保护区问题整改和违法违规参地还林工作。

　　多措并举保护生态系统。高标准实施山水林田湖草生态修复治理工程，完成生态红线划定，推进生态示范创建，加强各类保护区和湿地建设，构建优质生态廊道和生物多样性保护网络，争创国家级山水林田湖草综合发展示范区、全国土地节约集约模范市、国家级绿色矿业发展示范区。创新建设地质灾害监测预警平台，利用卫星遥感、大数据等手段对全市157个重点地质灾害点、旅游景区、边境公路实施全方位、全天候监测。

　　坚持不懈倡导绿色发展方式。实施企业节能降耗、节能产品惠民和气化白山工程，培育循环经济示范企业和项目，推广应用节能新技术，加快调整能源结构。严把生态环境准入关，严格限制高能耗高污染项目落地，探索实行新建项目排污总量减量、等量替代，从源头上减少污染。

　　（六）聚焦以人民为中心的社会治理方式，推动民生福祉高质量。坚守底线、突出重点，尽力而为、量力而行，让改革发展成果更多更公平惠及全市人民，办好39件民生实事。

　　坚决打好脱贫攻坚战。贯彻精准扶贫精准脱贫基本方略，完成减贫3460人，贫困村全部退出，贫困县全部摘帽。对全部建档立卡贫困户再摸底确认，瞄准特定贫困群众“一户一策”细化精准帮扶措施。加强督查巡察，完善长效机制，巩固提升脱贫成果，确保已退出的贫困村不滑坡、非贫困村不倒退、脱贫人口不返贫，切实做到真脱贫、脱真贫。

　　健全社会保障体系。全力筹措资金，坚决确保养老金按时足额发放。深入实施全民参保计划，加快完善城乡居民基本医疗等制度。健全社会救助机制，城乡低保和特困救助供养不低于省定标准。大力发展社会福利和慈善事业，坚决维护弱势群体合法权益。促进高校毕业生、去产能职工等群体多渠道就业创业，开展职业技能培训1万人次，城镇新增就业2.5万人，农村劳动力转移就业10.5万人。

　　大力发展社会事业。坚持教育优先，新改扩建7所幼儿园，实施“雨露计划”，推行中小学课后托管等服务，努力让每个孩子都能享受公平优质教育。深入推进健康白山建设，健全分级诊疗、现代医院管理和药品供应保障制度，推进公立医院薪酬制度改革，力争市中医院晋级三级中医院，全面提升妇幼健康服务水平。广泛开展竞技体育和全民健身，举办国际雪联越野滑雪中国巡回赛、全国越野滑雪锦标赛等赛事。扎实推进现代公共文化服务体系建设，乡镇文化站和农村文化广场覆盖率达85%以上。打造“原创音乐节”“农民村晚”等特色文化品牌，推出《天地长白》《走进长白山》等精品力作，努力满足群众高品位精神文化需求。

　　着力推动安全发展。严格落实党政领导干部安全生产责任制，健全风险分级管控和隐患排查治理双重预防体系，抓好煤矿、消防、道路交通等重点行业和领域专项治理，确保安全生产形势持续稳定好转。实现连续40年无重大森林火灾。进一步夯实监管责任、健全监管机制、完善监管体系、提升监管能力，全面加强食品药品等领域监管工作。强化综合防灾减灾救灾体系，有效提升应急处置能力。

　　深入推进平安白山建设。推动扫黑除恶专项斗争向全面纵深开展，系统提升“打伞断血”“反腐拍蝇”质效。充分发挥“雪亮工程”作用，构建信息化、智能化、亲民化社会治安防控体系，着力提升见警率、管事率。及时排查调处社会矛盾纠纷，巩固信访积案化解成果，切实解决群众合理诉求。严格履行地方金融监管职责，加强非法集资、互联网金融等重点领域风险防范处置，严厉打击违法违规金融活动，坚决守住不发生系统性金融风险的底线。深入开展双拥共建，促进军民深度融合发展。强化边境安全管控，推进兴边富民行动，确保边境繁荣和谐、人民安居乐业。

　　（七）聚焦改进作风提高执行力，推动政府工作高质量。深入推进全面从严治政，以新气象新担当新作为建设人民满意的服务型政府。

　　讲政治，把政治建设摆在首位。进一步树牢“四个意识”，始终在思想上政治上行动上同以习近平同志为核心的党中央保持高度一致。坚定“四个自信”，自觉用习近平新时代中国特色社会主义思想武装头脑、指导实践、推动工作。落实“两个坚决维护”，坚持把党对一切工作的领导贯彻到政府工作各个领域各个方面，确保党的路线方针政策得到全面落实。

　　讲法治，深入推进依法行政。坚持宪法至上，信仰法律、尊崇法律、践行法律，把政府一切活动纳入法治化轨道。严格规范重大行政决策行为，认真落实公众参与、专家论证、合法性审查等制度，有效提升依法科学民主决策水平。加强行政执法监督，严格规范公正文明执法。大力推进政务公开，确保政府权力在阳光下运行。认真落实市人大及其常委会作出的决议决定和审议意见，依法向市人大及其常委会报告重大事项。自觉接受人大、政协和社会各界监督，认真办理人大代表建议和政协委员提案。把诚信施政作为重要准则，有诺必践、有诺必行、有诺必果，以政府诚信引领社会诚信。

　　讲担当，狠抓工作落实。认真贯彻落实上级决策部署，推动各项工作任务项目化、责任化，做到纵向到底、横向到边，确保政令畅通、令行禁止。大力发扬钉钉子精神，一锤接着一锤钉，以披荆斩棘、逢山开路、遇水架桥的劲头，以愚公移山、精卫填海、蚂蚁搬丘的意志，有效打通政策落实“最后一公里”。倡导追求卓越，坚决防止工作不合格、不达标情况出现。大兴调查研究之风，深入基层一线，拿出具体措施，解决实际问题。完善绩效考评体系，建立大督查机制，有效解决干与不干、干好干坏一个样问题，加快形成比学赶超、争先进位的生动局面。

　　讲廉洁，持之以恒正风肃纪。严格贯彻中央八项规定和实施细则精神以及省、市具体规定，持续整治“四风”问题。加强源头治理，加大审计监督力度，严管公共资金、公共资源交易和国有资产资本，严控“三公”经费和一般性支出，用政府的“紧日子”换取群众的“好日子”。始终保持反腐高压态势，自觉接受监察委员会的监督，坚决查处腐败和损害群众利益行为，巩固不敢腐的氛围、完善不能腐的制度、构筑不想腐的堤坝，让清正清廉清明成为政府鲜明底色。

　　各位代表！新思想引领新时代，新使命开启新征程。让我们更加紧密团结在以习近平同志为核心的党中央周围，在省委、省政府和市委坚强领导下，不忘初心、牢记使命，团结拼搏、开拓奋进，为开创新时代白山绿色转型全面振兴高质量发展新局面而努力奋斗！
